# Needs and opportunities to future-proof crops and the use of crop systems to mitigate atmospheric change

**DOI:** 10.1098/rstb.2024.0229

**Published:** 2025-05-29

**Authors:** Stephen P. Long

**Affiliations:** ^1^ Institute for Genomic Biology and Departments of Plant Biology and of Crop Sciences, University of Illinois at Urbana-Champaign, Urbana, IL 61801, USA

**Keywords:** food security, rising CO_2_, drought, flooding, *Miscanthus*, rising temperature

## Abstract

Predicted changes in atmospheric composition and climate affecting crop productivity are reviewed. These include changes in both average conditions and extreme events, with respect to temperature, drought, flooding and surface ozone, coupled with rising atmospheric [CO_2_]. Impacts on, and means to adapt, crops to these changes are reviewed and outlined. Particular emphasis is given to (i) the results from open air field manipulations of surface atmosphere, temperature and soil water to understand impacts and adaptation and (ii) demonstrated genetic manipulations of photosynthesis and water use that could support future food supply under current and future conditions. Finally, attention is given to means by which crop systems could serve as CO_2_ collectors and carbon storage systems. Here, apparent opportunities are outlined for (i) manipulations of crops to enhance carbon storage and (ii) use of high-productivity sustainable perennial C_4_ grasses coupled with carbon capture and storage.

This article is part of the theme issue ‘Crops under stress: can we mitigate the impacts of climate change on agriculture and launch the ‘Resilience Revolution’?’.

*U.S. Secretary of State Anthony Blinken at the Global Solutions for Food Security Event in New York in September 2023 ‘if we don’t get this right, I actually don’t think anything else really, really matters’* [1].

## 1. Predicted future needs

Secretary Blinken’s remark truly underlines the gravity of our current situation and the seriousness of failing to address future food security. Recent and past history shows us that food shortages result in sharp price rises for any available food, leading to high levels of starvation, disruption of the social and political order of a country, and mass migrations [2–8]. By 2050−60 crops will experience a significantly different environment from today. Depending on location, a few changes will benefit crops, but the majority will lead to yield losses unless crops are future-proofed to anticipated change. Atmospheric [CO_2_] reached 427 p.p.m. in 2024 and is projected to be approximately 600 p.p.m. by 2050−60 [9,10]. This assumes the ‘representative concentration pathway’ (RCP) 8.5 of continued high emissions. With lower emissions, the concentration increase would of course be lower. However, actual increases have tracked RCP 8.5 for the past 20 years, with little sign of deviation [10]. This is underlined by the fact that the period from 2022 to 2024 saw the largest two-year jump in atmospheric [CO_2_] of any time since records began [9]. In June 2023, the average global temperature relative to the pre-industrial level, exceeded 1.5°C, and it remains above 1.5°C [11,12]. Yet, 1.5°C was the limit of warming in the Paris Agreement, a legally binding international treaty on climate change. It was adopted in 2016 by 194 individual countries, plus the EU, following the UN Climate Change Conference (COP21) [13]. Its failure, within just seven years from signing, signals a clear lack of commitment to addressing the problem with any urgency. Given the apparent absence of sufficient political will, RCP 8.5 (SSSP5-8.5 in the IPCC AR6 report [10]) is used here to address what may well come to pass by mid-century. While we may hope for a better scenario, future-proofing of cropping systems should prepare for the worst, as essential insurance.

Under this scenario, the global average temperature will rise another 1.2°C by 2050−60 to 2.7°C above pre-industrial temperature. This will be accompanied by more extreme temperature events. For example, in eastern Europe, the number of days in which 35°C is exceeded is predicted to quadruple from around 10 days in 2023 to 40 days. In the Caribbean, the number of days in which the temperature exceeds 41°C could rise from around 50 to 200 [10]. Precipitation will increase as a global average by 6%, but this will vary greatly between regions. It will very likely increase at high latitudes and in monsoon climates and likely decrease over large parts of the subtropics [10]. However, increased temperature and surface to atmosphere water vapour pressure deficit (VPD) will increase evapotranspiration and lower water use efficiency (WUE) [14]. Therefore, despite more precipitation, annual mean total column soil moisture is predicted to decline in most of South America, the central plains of North America, southern Africa, the Mediterranean and much of China and Australia, while increasing in equatorial Africa, central Asia and most high latitude regions. Precipitation on the wettest day of the year and hence the likelihood of flooding and run-off are projected to increase in all locations by 10–40%, with the current one-in-ten-year heavy rainfall event becoming a one-in-three-year event and 30% more intense under the SSSP5-8.5 scenario [10].

Why is future-proofing crop production against these changes so important? In the absence of climate change, a meta-analysis of 57 detailed global food scenario quantitative projections showed that the world will need between 35 and 56% more food by 2050 [15]. This is driven by three global trends: increased food wastage as the proportion of the global population living in urban areas increases, increased consumption *per capita* of meat and dairy and a rising global population [16]. If steady increases in temperature and drought are added, the maximum projected demand rises up to 62% [15]. However, the increase in crop losses to extreme events (fires, heat waves, floods and extreme droughts) raises the production capacity needed to provide sufficient food and reserves by a further 15–20% [17]. These extreme events will cause regional food supply shocks increasingly as climate change progresses, requiring increased food reserves as a buffer against starvation in the affected areas [17,18]. In short, to provide sufficient food and reserves, it may be necessary to almost double the yield per unit of land in current use. If supply fails to meet demand, then expansion of the crop production footprint and more destruction of natural habitats—in particular tropical forests—is an inevitable consequence [19,20]. This would further exacerbate climate change and the current rapid rate of biodiversity loss [21]. The number going hungry rose from 541 million in 2017 to 731 million in 2023 [22], a trend that can only continue as growth in demand exceeds production. The following sections outline the impacts and potential mitigation of specific aspects of change in atmospheric composition and climate on cropping systems.

## 2. Rising atmospheric [CO_2_]

Open-air elevation of [CO_2_] using free-air concentration enrichment (FACE) technology has provided us with the most realistic data on the direct effects of future [CO_2_] levels on crops [23]. In the absence of other changes and stresses, yields of C_3_ crops are substantially increased, with modern elite cultivars of rice and soybean showing yield increases of approximately 30% with elevation of [CO_2_] to anticipated 2050−60 levels. C_4_ crops—maize and sorghum—do not show a yield increase, since they are already [CO_2_]-saturated at today’s already elevated [CO_2_] levels [24]. However, under drought conditions yields can be increased, since stomata of both C_3_ and C_4_ show reduced aperture and conductance in elevated [CO_2_], resulting in improved conservation of soil water [24]. Elevated [CO_2_] does not always benefit crop production in a drought environment. In years when soil moisture is high during crop establishment, elevated [CO_2_] can lead to poorer root development, which then impacts the crop if soil moisture is depleted during the reproductive stage [25]. While yield may be increased, quality is generally depressed, particularly the protein content of non-legumes [26]. However, there is wide variation in protein and mineral content within the germplasm of the major grain crops, suggesting that breeding could quite easily address this quality loss [24].

Atmospheric [CO_2_] averaged 200 p.p.m. during the 2 Myr prior to the Industrial Revolution, the period during which our crops’ ancestors evolved [27]. In November 2024, the level had reached 424 p.p.m., more than double the level of 2 Myr ago [9,27]. Half of this increase has occurred in just the past 50 years, a very short period for natural or breeder selection to afford adaptation to these elevated conditions [9]. Analysis of limitations to photosynthesis suggests that metabolic control has been altered by this elevation. In C_3_ crops, control has shifted to become predominantly ribulose-1:5-bisphoshate (RuBP) regeneration-limited from RuBP carboxylase-oxygenase (Rubisco) limitation at lower [CO_2_] levels [28]. This indicated that upregulation of proteins limiting RuBP regeneration could further increase yields under elevated [CO_2_]. Plastid sedoheptulose-1:7-bisphosphatase (SbPase) is one enzyme that has been shown to limit RuBP regeneration [29]. Consistent with this theory, transgenic upregulation of SbPase in both tobacco and soybean resulted in significantly greater increases in photosynthesis and productivity in FACE than under current atmospheric conditions [30,31]. In contrast, the elevation of [CO_2_] that has already occurred has shifted the metabolic control of photosynthetic CO_2_ assimilation in C_4_ crops from phospho-enol pyruvate carboxylase (PEPc) to Rubisco [32]. This inferred that the elevation of Rubisco would increase photosynthesis and productivity in C_4_ crops. Consistent with this theory, transgenic upregulation of Rubisco in maize, sorghum and sugarcane resulted in increased photosynthesis at current [CO_2_], but not at pre-industrial levels. This also resulted in increased productivity in greenhouse conditions and—for sorghum—in replicated four-row plot field trials [16,33,34]. While these modifications that have increased productivity of both C_3_ and C_4_ crops under current and future conditions of elevated [CO_2_], they involved transgenesis. However, it is very likely that the same increases could be achieved by editing the promoter region of these genes, potentially avoiding or minimizing lengthy deregulation of such improved germplasm [16,34,35].

## 3. Tropospheric ozone

Tropospheric ozone (O_3_) is a secondary pollutant formed by the action of sunlight on volatile organic compounds and nitrogen oxides in polluted air masses. As these air masses drift into the countryside from the large urban areas in which they are formed, they continue to produce ozone in the presence of sunlight. These masses can drift across entire continents and even between continents. While reductions in fossil fuel use might lessen the problem, this will be likely counteracted by rising temperatures which enhance photochemical ozone formation. [O_3_] levels of ≥40 ppb can be damaging to crop productivity [36]. Pre-industrial levels are considered to have been about 5−15 ppb [37]. Today, levels of >100 ppb can be frequently found in rural areas of the US corn belt, with significantly higher levels in the major crop production areas of China and India [38,39]. A regression analysis of historical yield, climate and [O_3_] data for the USA was used to determine the losses resulting from [O_3_] for maize (*Zea mays*) and soybean (*Glycine max*) between 1980 and 2011. In rain-fed fields, this showed a loss owing to ozone of approximately 5% for soybean and approximately 10% for maize, costing some $9 billion annually [40]. Globally, losses of the major food crops could be in the region of 10% and rising [39]. Overcoming this would provide a substantial piece of the increased food supply needed. A key question is how future conditions of higher temperatures and drought will interact with O_3_ damage. While higher temperatures and drought generally coincide with higher surface [O_3_] levels, a 15 year open-air fumigation study showed that drought and elevated temperatures did not alter the vulnerability of soybean to a given level of ozone [41].

Ozone effects its damage on the plant by entering the leaf via the stomata, where it dissolves into the apoplastic fluid, producing an array of damaging active oxygen species [42]. An early downstream response is the loss of Rubisco [43]. Rising [CO_2_] means that leaves could assimilate the same amount of CO_2_ with lower stomatal conductance. Engineering or selecting for lower stomatal conductance would also decrease O_3_ uptake and damage, while upregulating Rubisco content could also lessen its impact. Antioxidant systems can remove the damaging oxidizing radicals produced from O_3_ absorption in the leaf [43]. Upregulation of these would also lessen ozone damage. Studies of the impacts of O_3_ on a range of maize, wheat and rice collections have revealed considerable variation in O_3_ tolerance and enabled the identification of loci associated with ozone tolerance and susceptibility [44–46]. This suggests a means for marker-assisted introgression of alleles conferring O_3_ tolerance to these major food crops.

## 4. Rising temperature

Rising temperatures will make conditions better or worse for crop production, depending on location. In the cool temperate zone, more frost-free days allow a longer growing season, facilitating the planting of longer season maturity types or allowing double cropping, such as winter cereal followed by soybean or a spring cereal within a single year. It will also allow the cultivation of crops at ever-higher latitudes, provided those locations have adequate soil quality for these crops. However, higher temperatures may also benefit weeds, pests and diseases. In addition , while growth and development may be accelerated, higher temperatures will reduce the length of the grain/seed-filling window, leading to lower yields. Crops that require insect pollination will suffer if rising temperatures cause a disparity between flowering and the pollinators’ period of abundance. On balance, with a further 1.2°C temperature rise by mid-century, yields are projected to be reduced by 9% (wheat), 16% (rice) or 5% (soybean) for current cultivars. In the tropics and warm temperate zone, increased frequency of extreme heat will likely increase incidence of crop failures, particularly when combined with drought [17]. The frequency of years when temperatures exceed thresholds for damage during critical growth stages will increase in all regions.

Ambient temperatures have a profound effect on plant growth, influencing all stages of development from germination and shoot growth to flower, fruit and seed formation, affecting both crop quality and yield. Higher temperatures can impair crop yields by decreasing photosynthesis, increasing respiration, and affecting development, pollination, fertilization and fruit/seed development. Higher temperatures will also mean higher leaf to air VPDs. The water vapour saturation pressure of air rises exponentially with temperature. Very few studies of elevated temperature have controlled VPD and the drying stress that it drives. As such, it is impossible in most cases to separate whether damage is a direct effect of temperature or indirect due to increased VPD [47].

In the absence of other stresses, the optimum temperature for crop photosynthesis is typically about 25°C for C_3_ crops and 35°C for C_4_ crops. Increases in temperature beyond these optima result in losses in photosynthesis, with a steep decline when the optimum is exceeded by about 10°C. In maize, photosynthesis declines by 50–60% at 40°C [48]. Across crops, this has been associated with a sharp decline in the activity of Rubisco activase (Rca), which serves to remove inhibitors from Rubisco. Clearly, there are wild species that can tolerate considerably higher temperatures, such as desert plants, and they may prove a source of more thermotolerant forms or inform edits that could improve the thermostability of Rca. Even within the germplasm of individual crops, variation in Rca temperature tolerance has been identified, including single residue changes that would improve thermal stability [49–51]. Small heat-shock proteins (HSPs), which serve to protect photosynthetic proteins, are induced by high temperatures. Upregulation of these HSPs may increase thermal tolerance of photosynthesis and other physiological processes [52].

Crops are generally more sensitive to elevated temperature during reproductive growth. Temperatures in excess of 35–39°C during this phase of growth generally lead to large or complete crop losses. In maize at ≥30°C, endosperm cell and amyloplast division are slowed, affecting sink strength and kernel size [53]. Pollen viability decreases at ≥35°C. Duration of pollen viability after anthesis is strongly dependent on moisture content, which in turn is strongly affected by the large elevation of VPD at these high temperatures. The result is that pollen viability will be low or absent by the time silking occurs [48]. While vegetative growth in soybean has an optimum temperature of 30°C, the optimum temperature for reproductive growth and development post-anthesis is just 23°C. An increase in the mean temperature above 23°C causes declines in seed growth rate, seed size and partitioning of biomass to grain (harvest index), reaching zero at 39°C. The viability of soybean pollen begins to decline at 30°C, with complete failure at 47°C [48]. Significant variation in pollen viability and pollen tube development was found in a survey of 44 genotypes [54], suggesting that with a wider search of germplasm, including the wild ancestor *Glycine soja* whose distribution extends from Siberia to sub-tropical China [55], better tolerance could be found. Rice similarly shows an optimum for vegetative growth of 33°C, but 25°C for grain development. The yield declines by about 10% for each 1°C above 25°C until 35°C, where yield is zero. This is associated with reduced pollen viability and production at temperatures above 25°C [48]. Significant genotypic variations in heat tolerance for percent filled grains, pollen production, pollen shed and pollen viability were found respectively across 14, 3 and 4 rice genotypes/ecotypes for heat tolerance, with greater pollen heat tolerance found in upland genotypes [56–58]. This suggests that a much wider search coupled with genome-wide association analysis would likely uncover considerable variation and associated loci that may be used in increasing reproductive viability at elevated temperatures. Of these major food crops, wheat shows the lowest optimum temperature for grain development, at just 15°C. This is primarily owing to the shortening of the grain filling window as temperatures increase, even in the absence of any reduction in photosynthesis. The increase in mean temperature during grain filling by each degree Celsius above 15°C lowers wheat yield by 6% [59]. A survey of 304 diverse elite winter wheat showed significant variation in heat stress tolerance of grain filling, identifying lines of potential value for breeding future-proofed wheat [60].

## 5. Drought and water use efficiency

By 2050, global yield losses to drought in maize are projected to rise to 21.3% from a previous average of 12.0% for the period 1961–2006, and for wheat from 9.6% to 15.5%. Of the areas of current crop production, the proportion that are drought-affected will rise most in Africa and Oceania, from the present 22 and 15%, respectively, to 59 and 58% by the end of the century [61]. Considerable effort was expended in identifying potential drought tolerance genes through screening of *Arabidopsis* mutants and relatives, which greatly expanded our understanding of the molecular responses of this plant to drought [62–64]. However, these have not translated into increased drought tolerance in crops, to date. The introduction of the *Bacillus subtilis* cold shock protein B (cspB), which downregulates ethylene production in drought, into maize appears to have improved drought tolerance and yield [65]. Two years of trials in Nigeria showed a significant yield increase under moderate drought conditions [66]. RNAi suppression of farnesyltransferase increased the Abscisic acid (ABA) sensitivity of guard cells and in turn, the drought tolerance of canola (*Brassica napus*), improving yields by 26% in field trials [65,67]. Despite these advances and other promising biotechnological approaches [65], these innovations have not been widely deployed.

Drought tolerance could also be improved by increasing crop WUE. Higher WUE results in a crop depleting soil water reserves more slowly, which can increase survival of transient droughts. Globally, 71.3% of freshwater withdrawals in 2020 were used in agriculture, primarily for irrigation. Despite declining freshwater availability, the proportion allocated to agriculture continues to rise. For example, India’s freshwater use in agriculture doubled between 1975 and 2010 as its population and food demand rose. This trend has continued: 32% of agricultural land in India was irrigated in 2001, rising to 42% in 2020 [68]. In 2020, 21% of global cropland was irrigated and this land accounts for about 40% of total food production [68,69]. Even without any increase in future food demand, more currently rain-fed land would need to be brought into irrigation to maintain current yields. This is because rising VPD will lower WUE [14,47]. Given our dependence on irrigated land, it is hard to see how a 60+% increase in food supply could be achieved within existing water resources. Solutions will be in more efficient use of irrigation water and bioengineering/breeding of more water use-efficient crops.

One opportunity results from rising [CO_2_]: as [CO_2_] rises, the same rates of assimilation can be achieved with lower stomatal conductance. Plants do respond to increased [CO_2_] with partial stomatal closure, but this is insufficient to take full advantage of the elevated [CO_2_] in the atmosphere. This is particularly the case for C_4_ crops, where photosynthesis is already [CO_2_] saturated in the current atmosphere [16]. We therefore need to engineer or breed for decreased stomatal conductance to obtain the improved WUE that can be achieved under rising [CO_2_]. Over-expression of Photosystem II Subunit S (PsbS) lowered the redox state of Q_a_, the primary electron acceptor of Photosystem II, at all light levels, without affecting the CO_2_ assimilation rate but lowering stomatal conductance. The result was a 15% improvement in leaf-level WUE in field-grown tobacco and a 30% decrease in whole plant water use [70,71]. An alternative approach to affecting stomatal behaviour is to reduce stomatal density (SD). Elucidation of the genes controlling steps in stomatal development has allowed targeted transformation and edits to decrease SD [72]. Moderate reductions (<50%) in SD by transgenic expression of epidermal patterning factor (EPF) genes in both rice and wheat improved WUE by approximately 15–20% without affecting yield [73,74]. Similarly, moderate reductions in SD by constitutive expression of a synthetic EPF transgene in C_4_ sorghum lowered plant water use by approximately 15% without affecting CO_2_ uptake [75]. However, substantial variation in SD across the germplasm of different crops is likely. For example, SD varied 2.5-fold across 235 rice accessions [76]. By combining optical topometry and machine learning, a high-throughput phenotyping method has become available to rapidly screen large amounts of germplasm for variation in SD and other morphological properties of the stomatal apparatus. This will hugely shorten the time needed to screen large collections of germplasm and would also aid phenotyping in the introgression of lower SD into elite cultivars [77]. Another factor affecting WUE is the speed of adjustment of stomatal conductance to fluctuations in light. Within crop canopies light is continually fluctuating; on transfer of leaves from sun to shade, CO_2_ assimilation drops abruptly, but the stomata require many minutes to adjust to the greatly reduced flux. As a result, leaf WUE is low during this period of adjustment. Across the leaf canopy and the day, this amounts to a considerable lowering of crop WUE in the field environment [78–80]. There is again considerable variation in the speed of this adjustment within the germplasm of individual crops: for example, WUE during a light transient across 15 contrasting accessions of rice differed twofold [81].

## 6. Flooding

Ironically, just as the frequency, duration and intensity of drought events are predicted to increase, so is flooding. After droughts, flooding is the second largest cause of crop losses, accounting for 19% of global losses between 2008 and 2018 [82]. Flooding affects crop production by preventing planting or by causing plant mortality after planting by starving the roots of oxygen. Water itself is of course not toxic, but diffusion of gases—including oxygen—is some 10 000 times slower in water than in air, leading to rapid depletion of oxygen in flooded soils. Therefore, on flooding, soils rapidly become anaerobic, i.e. have insufficient oxygen for aerobic respiration. In the absence of oxygen as the terminal acceptor of mitochondrial electron transport, ATP supply is greatly diminished. In addition, soil bacteria seeking alternative electron acceptors will reduce many ions to forms that are toxic to the roots. For example, sulfates and sulfites are reduced to hydrogen sulfide, which is highly toxic to plants, although it is also a key signalling molecule for acclimation at low concentrations [83]. Wetland plants, including rice, avoid these problems by producing roots with aerenchyma, which are large air spaces within the root cortex that permit oxygen from the shoot to reach the roots. This allows them to function normally and produce an encasing oxidized soil layer that will re-oxidize toxic ions formed under the anaerobic conditions of the surrounding soil. Could this strategy be incorporated into the other major crops? A survey of the root anatomy of 256 different maize cultivars and relatives showed a tenfold variation in the proportion of root aerenchyma. Germplasm in which aerenchyma occupied more than one-quarter of the cross-sectional area, equivalent to that found in marsh plants, has been identified [84]. Defence against hypoxia on flooding of soils can also be induced. This shows considerable potential for breeding tolerance to flooding of soils. Hypoxia triggers ethylene biosynthesis, which acts as a signal to promote both antioxidant activity and aerenchyma formation [85]. However, in most current crop cultivars this is insufficient to avoid damage and loss. Understanding of the molecular basis of adaptive plant responses to soil hypoxia has been greatly advanced in recent years [86,87], opening an opportunity to upregulate these responses to protect roots during flooding. Root aerenchyma does not help if flooding is so severe that the shoot is also submerged and starved of oxygen. This can be a frequent occurrence for rice, where 35% of land planted to rice is prone to floods that can submerge the developing crop for days or weeks [88]. Here, however, remarkable progress has already been made. Surveying the ability of rice cultivars to survive up to two weeks of complete submergence, a tolerant cultivar was discovered and associated with an allele *Sub1A-1* at the *Submergence 1* locus. This gene is rapidly induced on submergence in contrast to other alleles, and allows the plant to survive. Subsequent introgression of *Sub1A-1* into flooding intolerant cultivars made them flooding tolerant [89]. This discovery then allowed marker-assisted introgression of *Sub1A-1* into a wide range of germplasm, particularly aiding poor farmers in the most flood-prone areas, who rapidly adopted the flood-tolerant lines [90].

Most potential solutions, as outlined above, have concerned single key variables of global change. Attention must also be given to atmospheric and climatic changes that include a combination of two or more stress factors, such as drought and heat. These could subject plants to complex conditions of stress combination that require additional molecular discovery and breeding efforts [24,25,41,91–93].

## 7. Modifying crops for carbon sequestration

The annual net flux of carbon dioxide into the terrestrial biosphere was estimated for the period 2010-19 to be 5.3 Gt (C) [10]. Arable crops occupy 1.7 billion acres worldwide. Today, for our major crops, about 50% of the above-ground biomass is harvested as grain or seed, and the remaining 50%—stem, leaves, chaff and other waste—can remain on the field and may be incorporated into the soil. A further significant fraction of biomass is root, which remains within the soil. Maize, rice, soy and wheat are the top four arable crops in terms of land area occupied: 721 Mha in 2021. Their aggregate average yield was 4.3 t ha^−1^ in 2021 [94]. If we assume that the same amount of mass remained on this land area after harvest, plus another 50% as roots, then this would amount to 2.7 Gt (C) for just these four crops, assuming C is 40% of the dry biomass. Of course, this has little value as a carbon sink if this residue is decomposed within a few years, but if it could be kept in the soil, this would increase the net terrestrial sink by 50%. While deep tillage of agricultural soils created a past major source of CO_2_ to the atmosphere, modern agronomic methods have begun to reverse losses of carbon from soils. No-till or minimal till soil cultivation have been facilitated by herbicide resistance traits and have resulted in some carbon accumulation [95,96]. How do we now retain substantially more carbon in the soil? Two opportunities in combination would allow this: modifying crop residues to be more recalcitrant to decomposition processes, so slowing return to the atmosphere, and larger and deeper root systems [97]. What is the opportunity and likelihood of this?

Increasing the amount of aerenchyma in roots, besides providing protection against flooding, also allows the development of more roots, including deeper roots, without requiring more investment in tissue mass. Typically, the water content of the soil increases with depth and the lower layers may be anaerobic. Aerenchyma will allow the root to function and gain water and nutrients in these conditions [98], but on death, this root biomass will decompose far more slowly under anaerobic conditions than in the higher, more aerobic soil layers [99]. Deep root remains are also well below the depth of tillage [99]. Considerable genetic variation exists in the size, depth and architecture of root systems that could be exploited to increase both the efficiency of resource capture and soil carbon sequestration [84,100–102]. In parallel, factors controlling root architecture, rooting depth and root mass are being revealed at the molecular level [103–107].

A massive effort has been placed into understanding what makes plant cell walls difficult to deconstruct and how to bioengineer walls that can be more easily deconstructed for the efficient production of cellulosic biofuels [108–111]. This has also taught us how to make cell walls more recalcitrant so that crop residues will form a more effective soil carbon sink. From this prior work, it can be deduced that increased lignin content increases the recalcitrance of cell walls. Lignin is a random cross-linked polymer of three major monomers referred to as p-hydroxyphenyl (H), guaiacyl (G) and syringyl (S). Enrichment of the H content is shown to decrease polymerization and in turn recalcitrance in *Arabidopsis*. Conversely, lowering the H content should increase recalcitrance. Hydrophobic components of cell walls and surfaces are particularly recalcitrant. Plant cuticles consist primarily of cutin C32−36 polymers of C16 and C18 esterified and oxygenated fatty acids with small amounts of phenyl-propanoids [112]. They are by far the longest persisting components of plant remains in soils, typically being the only fossil remnants surviving thousands and millions of years [113]. Suberin is similarly hydrophobic, consisting of dimethyl esters of epoxyoctadecanedioic acids, with different levels of hydroxylation [114]. Decomposition of both cutin and suberin in soils is greatly slowed when complexed with lignin [115]. These findings suggest that to preserve a much higher proportion of root and crop residue in the soil would best be achieved by increasing the proportion of cutin and suberin, together with increased lignin of lower H content.

## 8. Perennial C_4_ grasses for atmospheric CO_2_ capture

Afforestation has been widely viewed as a means to offset carbon emissions. Forests, in their aggradation stage, may sequester about 3.2 t [CO_2_] per hectare into new biomass per year [116]. A mature crop of the perennial C_4_ grass, *Miscanthus × giganteus* (*Miscanthus*), grown in Illinois, sequestered 130 t [CO_2_] per hectare in one year [117]. As productive as this may seem, plant breeders have identified substantially more productive *Miscanthus* genotypes [118]. *Miscanthus*, in common with other highly productive C_4_ perennial grasses, such as switchgrass (*Panicum virgatum*) and prairie cordgrass (*Spartina pectinata*), produce an annual crop of shoots from perennating rhizomes [119,120]. In the autumn, the shoots senesce and translocate nutrients to the rhizome, which is then used by next year’s crop of shoots the following spring [121]. The stems are harvested on completion of dry-down in the autumn. This makes these crops highly sustainable and able to produce biomass with very few or no inputs following establishment; further, they add large amounts of organic matter to the soil through the turnover of their large root and rhizome systems, which also serve to bind the soil [117,121]. The perenniality and sustainability of these crops make them particularly suitable to lands that are marginal for arable production because of their susceptibility to erosion or because soil quality is insufficient for the economic production of food crops. The performance of the three perennial grasses *Miscanthus*, switchgrass and energycane (*Saccharum officinarum* L. hybrids - fibre crops derived from sugarcane breeding) was assessed for marginal land capable of producing a crop under rain-fed conditions in the eastern USA. Yields were predicted to high spatial resolution based on gridded soil data and down-scaled current and future climate at each location. Selecting the most productive of these three crops for each location, it was estimated that these crops on this marginal land would remove 600 million tonnes of CO_2_ from the atmosphere into harvestable shoot biomass per annum [118]. Much of the geology of the eastern USA is suitable for deep CO_2_ storage. Using BECCS (bioenergy with carbon capture and storage), this biomass could be burnt to generate electricity and the resulting CO_2_ captured and transferred to deep underground storage [118]. This CO_2_ removal statistic does not take account of the additional benefits of displacement of fossil fuel use in electricity generation or the carbon that will accumulate in the soil under these crops. Such large-scale plantings would alter surface albedo and latent heat transfer to the atmosphere sufficient to cause a regional summer cooling of 1°C, which would significantly offset regional climate change-induced warming [122]. There are also key opportunities to similarly utilize somewhat less productive but drought tolerant desert perennials on degraded hot semi-arid lands [123–126].

## 9. The key role of plant breeding

The above sections have outlined many potential opportunities for adapting crops to future global change and even using crops to remove CO_2_ from the atmosphere. Many of these opportunities require incorporating transgenes or introgression of advantageous alleles from crop relatives. While the acceptability of transgenic crops is slowly rising, particularly where it is most needed, the pathway to deregulation, where allowed, is still a long and expensive one [127,128]. Two developments, however, could greatly accelerate this process. In many cases, transgenic improvements involve upregulation by adding extra copies of a gene that the crop already has. This can now likely be achieved by editing the upstream non-coding region of the gene to, for example, remove repressor elements [35,129]. Such scarless editing is increasingly being accepted as non-transgenic [130]. Rapid progress in functional simulation of key proteins is allowing *in silico* testing of multiple amino acid residues to predict, for example, more efficient forms of key enzymes [131]. Again, these changes could be achieved by scarless editing, avoiding the need for transgenic upregulation.

**Figure 1.**
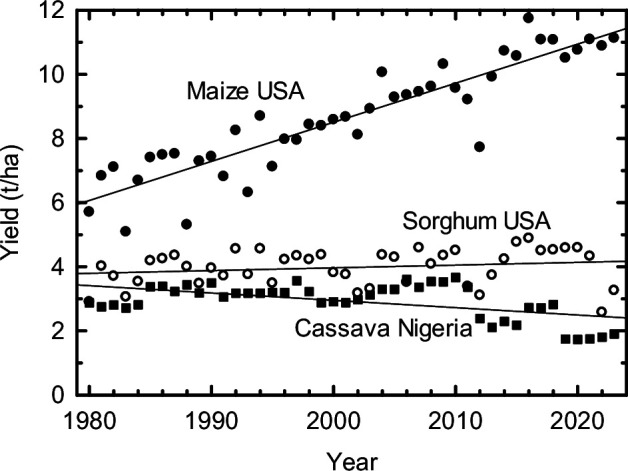
Average yields of maize and sorghum in the USA and cassava in Nigeria. Cassava is given as dry mass, assuming it has a moisture content of 70%. Data from [137].

Key to achieving any of these opportunities are the roles of plant breeders and seed systems. These changes can only be implemented by introgression of these into regionally adapted and accepted cultivars and identifying the genetic backgrounds in which the transgene, edit or allele from a crop relative is most effective [132]. Unfortunately, introducing just the opportunities outlined in the previous sections appears far beyond the current capacity for plant breeding. Just as plant molecular biological opportunities have mushroomed, the capacity of public domain plant breeding to implement these innovations has declined dramatically. The past four decades have seen a sharp decline in public plant breeding, offset to some extent by the ascendency of a few multinational companies with vast plant breeding capability [133,134]. Unfortunately, most of this multinational effort must concentrate on short-term financial gain, which has been best served by a focus on North American hybrid maize improvement [135]. However, nearly 80% of maize produced in the USA is used for ethanol production or as animal feed [136], thereby contributing relatively little to global food security. Between 1980 and 2024, US maize yields doubled while sorghum improved by just 12% (figure 1). Maize improvement has largely resulted from massive investment by multinationals, while sorghum improvement has largely depended on depleted public domain efforts. Cassava, a major staple of much of Africa, showed no improvement in its average yield in Nigeria, the largest producer [137]. This is despite progress in breeding to counter increasing losses owing to pests, diseases and drought (figure 1) [138,139]. The example of maize in the USA shows what could be achieved with other crops in the public domain with similar investment. Although now aided with high-throughput phenotyping tools, marker-assisted and genomic breeding, it is hard to see how the opportunities for future-proofing our crops that are highlighted in this review and in accompanying articles can be implemented at the scale that is necessary. The discovery of the rice *Sub1A-1* allele, its introgression into a wide range of rice cultivars and subsequent availability through seed systems provide a shining example of what can be achieved in the public domain for some of the world’s poorest farmers [89]. A second emerging example of such success through collaboration is the approval of cowpea carrying the *Bacillus thuringiensis* (Bt) *cry1Ab* gene in Nigeria and now Ghana. This was the first transgenic food crop approved for distribution to farmers in Africa, excepting South Africa’s acceptance of bt maize. Its adoption by farmers prevents the devastating losses caused by the bean pod borer, *Maruca vitrata,* which can cost up to 90% of this key staple for the region [128,140]. These examples show the success that can be achieved when molecular biology, breeding and seed distribution systems work together for a common cause. But many more such examples will be needed if we are to avert famines on a scale not seen for decades. Achieving this requires the training of more plant breeders in both national and international programmes and access to relevant high-throughput phenotyping and genotyping facilities, especially in the countries that will continue to be most affected by food shortages. It also requires much swifter and more efficient deregulation of key biotechnologies. While there is still time, these needs deserve our most urgent attention.

‘Some see things as they are, and ask why. I dream of things that never were, and ask why not?’ Robert F. Kennedy

## Data Availability

This article has no additional data.
